# Anaerobic poly-3-d-hydroxybutyrate production from xylose in recombinant *Saccharomyces cerevisiae* using a NADH-dependent acetoacetyl-CoA reductase

**DOI:** 10.1186/s12934-016-0598-0

**Published:** 2016-11-18

**Authors:** Alejandro Muñoz de las Heras, Diogo J. Portugal-Nunes, Nathasha Rizza, Anders G. Sandström, Marie F. Gorwa-Grauslund

**Affiliations:** 1 Division of Applied Microbiology, Department of Chemistry, Lund University, PO Box 124, 221 00 Lund, Sweden; 2 Vattenhallen Science Center, John Ericssons väg 1, 223 63 Lund, Sweden; 3 Novozymes A/S, Krogshoejvej 36, 2880 Bagsvaerd, Denmark

**Keywords:** *Saccharomyces cerevisiae*, Poly-3-d-hydroxybutyrate (PHB), Xylose, NADH, NADPH, Acetoacetyl-CoA reductase

## Abstract

**Background:**

Poly-3-d-hydroxybutyrate (PHB) that is a promising precursor for bioplastic with similar physical properties as polypropylene, is naturally produced by several bacterial species. The bacterial pathway is comprised of the three enzymes β-ketothiolase, acetoacetyl-CoA reductase (AAR) and PHB synthase, which all together convert acetyl-CoA into PHB. Heterologous expression of the pathway genes from *Cupriavidus necator* has enabled PHB production in the yeast *Saccharomyces cerevisiae* from glucose as well as from xylose, after introduction of the fungal xylose utilization pathway from *Scheffersomyces stipitis* including xylose reductase (XR) and xylitol dehydrogenase (XDH). However PHB titers are still low.

**Results:**

In this study the acetoacetyl-CoA reductase gene from *C. necator* (CnAAR), a NADPH-dependent enzyme, was replaced by the NADH-dependent AAR gene from *Allochromatium vinosum* (AvAAR) in recombinant xylose-utilizing *S. cerevisiae* and PHB production was compared. *A. vinosum* AAR was found to be active in *S. cerevisiae* and able to use both NADH and NADPH as cofactors. This resulted in improved PHB titers in *S. cerevisiae* when xylose was used as sole carbon source (5-fold in aerobic conditions and 8.4-fold under oxygen limited conditions) and PHB yields (4-fold in aerobic conditions and up to 5.6-fold under oxygen limited conditions). Moreover, the best strain was able to accumulate up to 14% of PHB per cell dry weight under fully anaerobic conditions.

**Conclusions:**

This study reports a novel approach for boosting PHB accumulation in *S. cerevisiae* by replacement of the commonly used AAR from *C. necator* with the NADH-dependent alternative from *A. vinosum*. Additionally, to the best of our knowledge, it is the first demonstration of anaerobic PHB synthesis from xylose.

**Electronic supplementary material:**

The online version of this article (doi:10.1186/s12934-016-0598-0) contains supplementary material, which is available to authorized users.

## Background

Most of today’s energy and materials are primarily coming from petroleum based resources that are non-renewable. Petroleum derivatives such as plastics are one example of an important commodity in contemporary society, with a world annual plastic production in 2014 of 311 million tons and that is estimated to double again in the next 20 years [1]. An alternative could be to use biopolymers that are produced in nature, for instance isoprenoids and polyhydroxyalkanoates (PHAs). Of the PHAs, poly-3-d-hydroxybutyrate (PHB) has been suggested to be one of the most promising biopolymers, as it shares many physical properties with polypropylene [2].

Several microorganisms are capable of accumulating intracellular granules of PHB through polymerisation of soluble molecules, hence preventing leakage of valuable compounds out of the cells. In both gram-positive and gram-negative bacteria, PHB is used as carbon storage when nutrient supplies are imbalanced [3]. *Cupriavidus necator* (formerly known as *Ralstonia eutropha*) is one of the most studied bacteria for its ability to accumulate considerable amounts of PHB [4]. The biosynthesis of PHB in this bacterium is catalyzed through three enzymatic reactions where the first reaction allow the condensation of two acetyl coenzyme A (acetyl-CoA) molecules into acetoacetyl-CoA by β-ketoacyl-CoA thiolase (encoded by *phaA*). The next step is the conversion of acetoacetyl-CoA to (*R*)-3-hydroxybutyryl-CoA catalyzed by the acetoacetyl-CoA reductase (AAR) (encoded by *phaB*). Lastly, the (R)-3-hydroxybutyryl-CoA monomers are polymerized into PHB by the polyhydroxyalkanoate synthase (encoded by *phaC*). All three enzymes required for PHB synthesis in bacteria are located in the cytoplasm of the cell where PHB granule accumulation takes place [5, 6]. Most characterized PHB-producers carry an NADPH-dependent AAR gene, whereas AAR with putative promiscuous cofactor utilization are only reported in few cases: for the halotolerant bacterium *Allochromatium vinosum,* formerly known as *Chromatium vinosum* [6], the anaerobic syntrophic bacterium *Syntrophomonas wolfei* [7] and the recently described AAR from the halophilic bacterium *Halomonas boliviensis* [8].

However the economic feasibility of PHB bio-production is dependent on using a cheap and highly available source of fermentable sugars, such as forestry and agriculture residues. During lignocellulosic biomass pretreatment, several inhibitory compounds are released [9], generating a harsh environment in which PHB-producing bacteria species are not well adapted. Therefore the industrial workhorse *Saccharomyces cerevisiae* that is outcompeting other cell factories for inhibitor tolerance [10, 11] has been explored as an alternative for the production of PHAs, possibly as a valuable side-product from the lignocellulosic ethanol anaerobic process. The first attempt to produce PHB in *S. cerevisiae* was from the available cytosolic 3-hydroxybutyryl-CoA derived from fatty acid β-oxidation pathway through the overexpression of *phaC* gene from *C. necator* [12]. Later, PHB production was detected from glucose by expressing the three bacterial genes *phaA, phaB* and *phaC* from *C. necator* [13, 14]. In addition the pool of cytosolic acetyl-CoA was increased in *S. cerevisiae* by overexpressing the alcohol dehydrogenase (*ADH2*), the acetaldehyde dehydrogenase (*ALD6*), the acetyl-CoA acetyltransferase (*ERG10*) and the *Salmonella enterica* acetyl-CoA synthetase variant (*acs* ^*L641P*^) and it resulted in improved PHB productivity from glucose [15]. Finally since a major fraction of sugars from lignocellulosic biomass may consist of xylose, PHB pathway has been introduced in *S. cerevisiae* engineered for xylose utilization, leading to PHB synthesis from xylose [16]. In the present study we demonstrate that PHB can be produced anaerobically, and in combination with ethanol, from xylose by cofactor shift through the introduction of the NADH-dependent AAR alternative from *A. vinosum* in recombinant *S. cerevisiae*.

## Methods

### Plasmids

Plasmids used in the study are presented in Table 1. For the construction of YIpAGS3, the AAR gene from *A. vinosum* (GenBank Accession No. YP_003442070.1) was codon optimised for *S. cerevisiae* at Eurofins Genomics (Ebersberg, Germany) and synthesized by Genescript (Piscataway, NJ, USA). The coding sequence was designed to be under the control of the constitutive promoter-terminator pair of the gene *TPI1* flanked by the restriction sites *Sac*I and *Spe*I. The custom-synthesized coding sequence was cloned on the integrative plasmid YIpAGS2 [16] generating YIpAGS3 (Additional file 1).

**Table 1:** Plasmids used in the study

Plasmids	Relevant genotype	References
YIpOB8	*pTDH3*-*XYL1*-*tADH1*; *pPGK1*-*XYL2*-*tPGK1*; *URA3*	[20]
YIpDR7	*pTDH3*-*XYL1*(N272D)-*tADH1*; *pPGK1*-*XYL2*-*tPGK1*; *URA3*	[20]
YIpAGS2	*YIplac128; pTEF1*-*PhaA*-*tTEF1; pGPM1*-*C. necator PhaB1*-*pGPM1; pTPI1*-*PhaC1*-*tTPI1; LEU2*	[16]
YIpAGS3	*YIplac128; pTEF1*-*PhaA*-*tTEF1; pGPM1*- *A. vinosum PhaB*-*pGPM1; pTPI1*-*PhaC1*-*tTPI1; LEU2*	This study
YIplac128	*LEU2*	[45]

### Strains, media and culture conditions

Yeast strains used in the study are listed in Table 2. Yeast strains were recovered, from 20% glycerol stocks stored at −80 °C, on solid YPD (10 g/L yeast extract, 20 g/L peptone, 20 g/L glucose, 15 g/L agar) for two days at 30 °C. Yeast cultures were grown in liquid YPD medium for 14–16 h, or less when required, at 30 °C and 180 rpm in an orbital shaker. Competent yeast cells were prepared and transformed according to the High efficiency transformation protocol [17]. Transformants were selected on solid Yeast Nitrogen Base (YNB) medium (6.7 g/L Yeast Nitrogen Base without amino acids (Becton, Dickinson and Company, USA) supplemented with 20 g/L glucose or xylose and 15 g/L agar). Leucine was added for complementation at a concentration of 30 mg/L when required.

**Table 2:** *Saccharomyces cerevisiae* strains used in the study

Strain name	Relevant genotype	References
TMB3043	CEN.PK2-1C; *gre3*Δ; *his3*::*pPGK1*-*XKS1*-*tPGK1*, *HIS3*; *tal1*::*pPGK1*-*TAL1*-*tPGK1*; *tkl1*::*pPGK1*-*TKL1*-*tPGK1*; *rki1*::*pPGK1*-*RKI1*-*tPGK1*; *rpe1*::*pPGK1*-*RPE1*-*tPGK1*; *ura3*, *leu2*	[19]
TMB4420	TMB3043; *ura3*::YIpDR7; *leu2*	This study
TMB4424	TMB4420; *leu2*::YIplac128	This study
TMB4425	TMB4420; *leu2*::YIpAGS3	This study
TMB4440	TMB3043; *ura3*::YIpOB8; *leu2*	[16]
TMB4443	TMB4440; *leu2*::YIpAGS2	[16]
TMB4444	TMB4440; *leu2*::YIplac128	[16]
TMB4445	TMB4440; *leu2*::YIpAGS3	This study

*Escherichia coli* strain NEB5-α (New England Biolabs) was used for sub-cloning of plasmid DNA. Heat shock competent *E. coli* cells were prepared according to the Inoue method [18] and transformed according to the supplier’s instructions. Transformants were selected on solid Luria–Bertani (LB) plates (5 g/L yeast extract, 10 g/L peptone, 5 g/L NaCl, 15 g/L agar, pH 7.0), supplemented with 100 mg/L of ampicillin, for 16 h at 37 °C. Cultures of transformed *E. coli* were recovered from 25% glycerol stocks stored at −80 °C and grown in liquid LB medium, supplemented with ampicillin 50 mg/L, for 14–16 h at 37 °C and 180 rpm in an orbital shaker.

Strain TMB3043 [19], an engineered strain overexpressing the non-oxidative pentose phosphate pathway for efficient pentose utilization, was used as background strain in this study. TMB3043 was transformed with the linearized vectors YIpDR7 or the YIpOB8 [20], generating the strains TMB4420 and TMB4440, respectively (Table 2). TMB4420 and TMB4440 were subsequently transformed with the linearized vector YIpAGS3 generating the strains TMB4425 and TMB4445, respectively. TMB4424 was obtained from transformation of TMB4420 with linearized YIplac128. The YIpDR7 and YIpOB8 plasmids were digested with Fast Digest *Eco*RV (Thermo Scientific), YIplac128 with Fast Digest *Eco*9I and YIpAGS3 with Fast Digest *Ppu*MI (Thermo Scientific, USA). Transformed strains TMB4425 and TMB4445 were validated by amplifying stretches of the integrated cassette by PCR, using extracted genomic DNA as template and primers PhaC1_f: 5′CATATTACAATAATGGCCACTGGTAAAGG3′ and PhaC1_r: 5′CATTCATTCTTCAGACTTATGCCTTTGCTTTCACATAC 3′.

### Enzymatic assays

Cells were cultivated in 50 mL conical centrifuge tubes containing 5 mL of YNB-glucose, at 30 °C 180 rpm overnight. At starting OD_620_ of 1.0 the cells were inoculated in the same media as described above and harvested during the late exponential phase. Whole-cell protein extract was obtained by treating the cells with Y-PER extraction solution (Pierce, Rockford, IL, USA) according to the manufacturer's instructions. The total protein concentrations were measured with a Bradford assay using Micro BCA™ Protein Assay (ThermoFisher Scientific, Waltham, MA, USA) and bovine albumin standard (ThermoFisher Scientific, Waltham, MA, USA). Absorbance was measured at 340 nm on a Multiskan Ascent (Thermo Electro Corporation, Finland) using 96-well plates (final volume of 250 µL, in three replicates). Acetoacetyl-CoA reductase kinetics was determined using 200 µM acetoacetyl-CoA as substrate, 10 µL of cell extract, 200 mM NADH or NADPH as cofactor, 100 mM MOPS pH 7.0 as buffering agent and 5 mM 2-mercaptoethanol as reducing additive agent. The measured initial rates were used to calculate specific activities (µmol/min mg total protein).

### Aerobic cultivations in shake flasks

Single colonies were pre-inoculated in 5 mL YNB-xylose (13.4 g/L YNB without amino acids, 50 g/L xylose, 50 mM pH 5.5 phthalic acid) in 50 mL conical centrifuge tubes at 30 °C, 180 rpm overnight. Cells were harvested during the exponential phase and inoculated in 250 mL baffled shake flasks containing 50 mL buffered YNB-xylose, at starting OD_620_ of 0.05 and set at 180 rpm at 30 °C. The growth rate was followed by measuring optical density at 620 nm with an Ultrospec 2100 pro spectrophotometer (GE Healthcare Life Sciences, Sweden). The measurements were made in technical duplicates. Cultivations were performed in biological duplicates.

### Oxygen-limited cultivations

Oxygen-limited cultivations were performed in 65 mL serum flasks containing 50 mL of buffered YNB-xylose. For oxygen limited growth, media was supplemented with Tween 80 (400 mg/L) and ergosterol (10 mg/L) and 7 mL of mineral oil on top. Temperature was maintained at 30 °C in a water bath and agitation with a magnetic stirrer was set to 180 rpm. Inoculated cells were pre-grown under aerobic conditions to allow biomass formation for starting at OD_620_ of 0.1. Batch experiments were performed in biological duplicates.

### Anaerobic batch fermentations in bioreactors

Fully anaerobic fermentations were performed in Multifors 1.4 L vessels (Infors, Switzerland) containing 1000 mL unbuffered YNB-xylose supplemented with Tween 80 (400 mg/L) and ergosterol (10 mg/L). Bioreactors were initially inoculated with freshly grown cells to a starting OD_620nm_ of 0.5. The cells used for the inoculum were grown aerobically as described above in 50 mL conical centrifuge tubes and then transferred to 250 mL baffled shake-flasks to get higher levels of biomass. The bioreactors were sparged with 0.1 vvm nitrogen at 30 °C and 200 rpm. Silicon antifoam RD emulsion (Dow Corning, USA) was added to avoid excess foam formation. The pH was kept at 5.5 with the addition of 3M KOH and 3M H_2_SO_4_ solutions. The experiments were run independently twice.

### Metabolite determination and cell dry weight analysis

Cultures were regularly sampled for metabolite analysis. Extracellular metabolite levels were measured from the sample supernatants with HPLC using a Waters (Milford, USA) system equipped with an Aminex HPX-87H column (Bio-Rad, Richmond, USA) that operates at 60 °C with a mobile phase of 5 mM H_2_SO_4_ and 0.6 mL/min flow rate. Concentrations of glucose, xylose, xylitol, glycerol, acetate and ethanol were calculated from an external standard calibration curve. Samples were analysed in technical duplicates. Cell dry weight was determined by filtering samples through a pre-weighed 0.45 µm membrane filter (Pall Corporation, New York, USA) The membranes were washed with ultrapure H_2_O and dried in a microwave oven at 350 W for 8 min. The final weight was measured after equilibration to room temperature in a desiccator. The measurements were made in technical duplicates. In the case of the bioreactor fermentation, biomass was measured at each time point.

### PHB quantification

The method used relied on the quantitative conversion of PHB to crotonic acid catalysed by hot concentrated sulfuric acid [21]. PHB content was analysed by harvesting 2 mL of culture by centrifugation for 5 min at 6000×*g*. Supernatant was carefully removed and the pellets were resuspended in ultrapure H_2_O, followed by centrifugation for 5 min at 6000×*g*. This step was repeated twice, then 0.5 mL of 99% sulfuric acid was added onto the pellets that were incubated at 95 °C for 1 h with open lids in a heat block (Grant QBD1, Grant Instruments, UK). The resulted solution was diluted 20 times by serial dilution and further analysed by HPLC using the same conditions as for the metabolite analysis. Commercially available PHB (#363502, Sigma-Aldrich) processed in parallel with the samples was used as control. Concentrations of crotonic acid were calculated from an external ten-point standard calibration curve. Samples were analysed in technical duplicates.

### Enzyme structure homology modelling

Protein structure models were generated with automated homology modeling using SWISS-MODEL [22] by submitting translated gene sequences. The translated AAR gene from *A. vinosum* (GenBank Accession No. YP_003442070.1) was submitted, using the *C. necator* AAR structure as designated template (PDB: 3VZP) [23].

## Results

### Selection of putative NADH-dependent acetoacetyl-CoA reductase

**Fig. 1:**
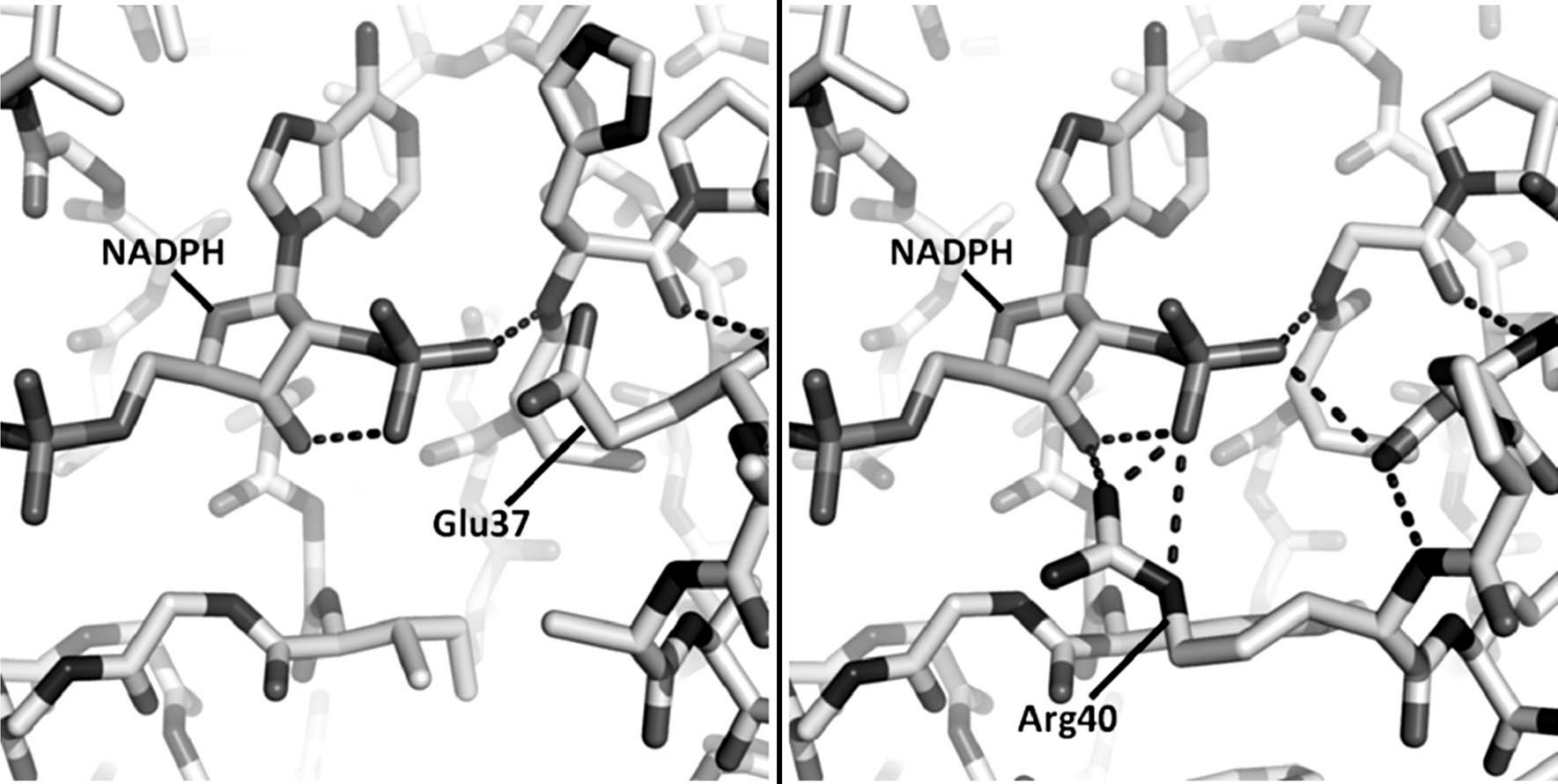
Cofactor binding sites of the AAR from *A. vinosum*, made through homology modeling (*left*) and the AAR from *C. necator* (PDB: 3VZP) (*right*). The cofactor NADPH is modeled in the binding site and Glu-37 is marked in the *A. vinosum* AAR, and the Arg-40, which forms several polar contacts to NADPH, is marked in the *C. necator* AAR

The AAR from *C. necator* uses NADPH as a cofactor [24], which competes with biomass synthesis in recombinant *S. cerevisiae* and could explain the low PHB titers observed on glucose and xylose [15, 16]. This led us to look for NADH-dependent alternatives in reported studies. Three AARs appeared to be promising candidates: the AAR from *A. vinosum* (GenBank: YP 003442070.1) that is a thiosulfate oxidizing, gram-negative bacterium accumulating PHB up to 58% of its CDW [25] and two AARs from *S. wolfei* species adapted for anaerobic synthropic growth [GenBank: ABI67978.1 (*Swol_0651*) and ABI69207.1 (*Swol_1910*)] [7, 26]. Since the crystal structures of these AARs were not available, protein structures were made with automated homology modeling using SWISS-MODEL [22]. The AvAAR displayed 56% protein sequence identity to the *C. necator* AAR whose crystal structure was used as template (PDB: 3VZP) [23]. The *S. wolfei* AARs had a protein sequence identity of 30% (GenBank ABI67978.1) and 36% (GenBank ABI69207.1) respectively, compared to the CnAAR. Both *S. wolfei* homology models (*Swol_0651* modelled on PDB: 4MOW [27] and *Swol_1910* modelled on PDB: 4DMM [28]) indicated a NADPH-binding arginine motif [23], typical of the CnAAR and related AARs. In contrast, the cofactor binding site of the AvAAR contained a dominant glutamate instead of an arginine (compared to the *C. necator* AAR) (Fig. 1). Acidic residues, such as glutamate in the case of the AvAAR, can create hydrogen bonds to the 2′- and 3′-hydroxyl groups on the adenine ribose of NADH [29], in comparison to arginine, which are ideally forming contacts to the 2′-phosphate in NADPH [23]. The AvAAR was also assumed to generate the correct enantiomer, (*R*)-3-hydroxybutyryl-CoA, preferred by the *C. necator* polyhydroxyalkanoate synthase for PHB accumulation as the binding site for the prochiral keto functionality on the acetoacetyl-CoA substrate was almost identical in the AvAAR homology model as in the CnAAR crystal structure. From the results above, the AvAAR was further used and evaluated in a recombinant PHB-producing *S. cerevisiae* host.

### Construction of NADH-dependent PHB pathways in recombinant xylose-utilising strains

**Fig. 2:**
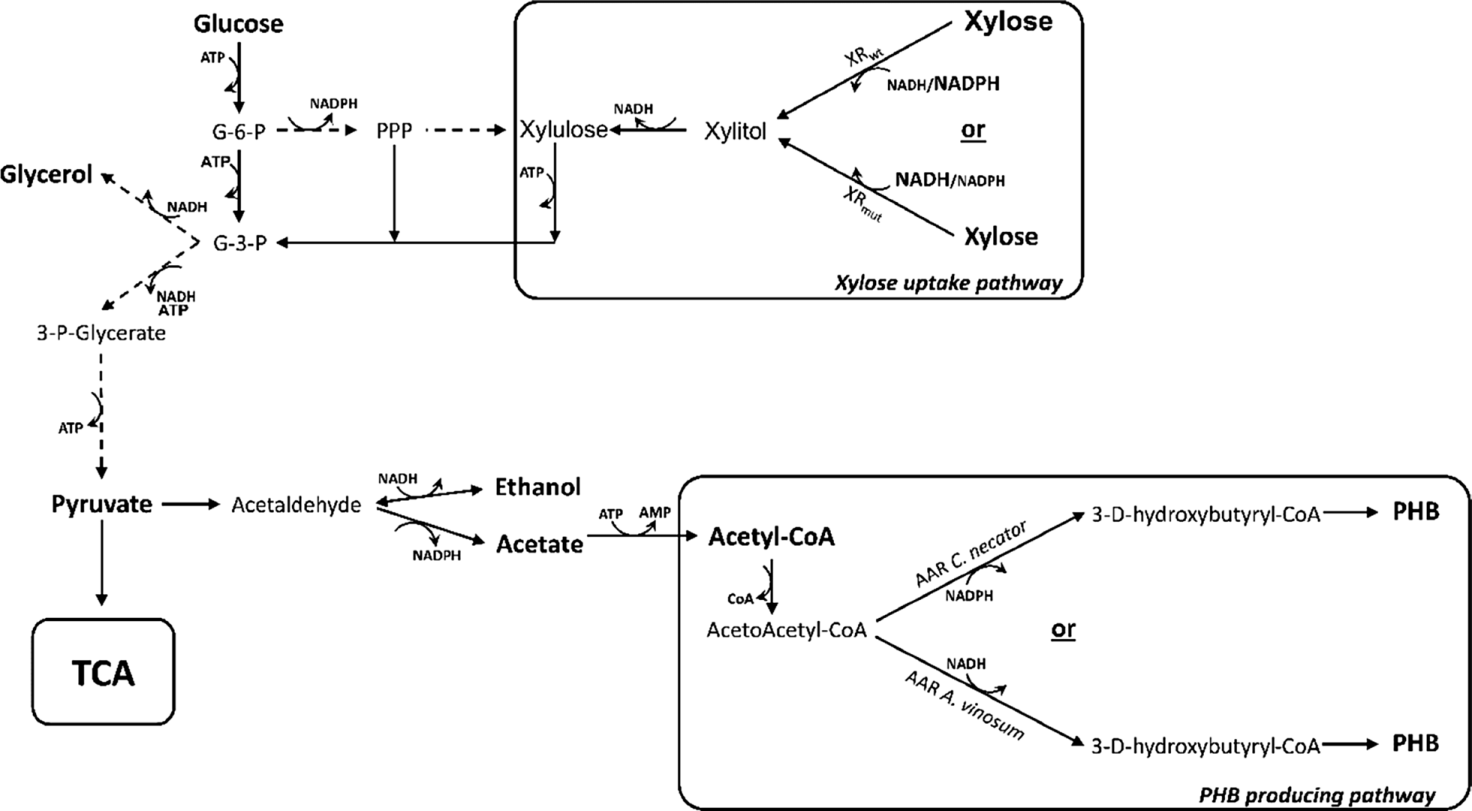
Metabolic diagram of the main central carbon metabolism including the XR and PHB pathway steps. *XR* xylose reductase, *NADH* nicotinamide adenine dinucleotide, *NADPH* nicotinamide adenine dinucleotide phosphate, *ATP* adenosine triphosphate, *AMP* adenine monophosphate, *TCA* tricarboxylic acid cycle, *PHB* poly-3-D-hydroxybutyrate, *AAR* acetoacetyl-CoA reductase, *Ac-CoA* acetyl coenzyme A, *G3P* glyceraldehyde 3-phosphate, *PPP* pentose phosphate pathway

The previously described PHB-producing xylose-utilizing *S. cerevisiae* strains carried the integrative yeast plasmid YIpAGS2 that contains codon-optimised genes encoding the β-ketothiolase, acetoacetyl-CoA reductase (AAR) and PHB synthase from *C. necator* under the control of constitutive promoters [16]. In this study YIpAGS3 (Table 1; Additional file 1) was constructed by replacing the AAR from *C. necator* (CnAAR) in YIpAGS2 with the codon-optimised homolog from *A. vinosum* (AvAAR). YIpAGS3 was then introduced in two xylose-consuming strain backgrounds: TMB4420 that carries a Asn272Asp substitution in the xylose reductase (XR_mut_) from *Scheffersomyces stipitis* [20] and TMB4440 that carries a wild type xylose reductase (XR_wt_) from *S. stipitis* [30]. These two alternatives were chosen because XR_wt_ is known to have a nearly exclusive NADPH-selectivity, consequently depletion of NADPH in the xylose reduction step is expected to impact both growth and NADPH-dependent PHB production in xylose-rich media [16, 20]. In contrast, XR_mut_ contains the N272D mutation in the cofactor binding that increases the enzyme specificity for NADH [20]. This is known to result in an improvement in the cofactor balance for xylose uptake (Fig. 2) that translates into enhanced anaerobic growth [20]. But the effect on PHB production is unknown.

The integrative empty vector YIplac128 and the newly constructed YIpAGS3, were used to transform TMB4420 (XR_mut_), generating strains TMB4424 (XR_mut_) and TMB4425 (XR_mut_, AvAAR), respectively. YIpAGS3 was also used to transform TMB4440 (XR_wt_), generating strain TMB4445 (XR_wt_, AvAAR). The strains were then compared with the control strain TMB4444 (XR_wt_) and with TMB4443 (XR_wt_, CnAAR) [16].

### In vitro activities of acetoacetyl-CoA reductases expressed in *S. cerevisiae*

In order to check whether the introduced *A. vinosum* AAR gene was functional in *S. cerevisiae*, enzymatic assays were performed in strains TMB4443 (XR_wt_, CnAAR) and TMB4445 (XR_wt_, AvAAR) carrying *C. necator* and *A. vinosum* AAR, respectively (Table 3).

**Table 3:** Acetoacetyl-CoA reductase (AAR) specific activity

Strain	Acetoacetyl-CoA reductase	Cofactor	V_max_ (µmol/min mg total protein)
TMB4443	*C. necator*	NADH	ND
		NADPH	9.9 ± 3.9
TMB4445	*A. vinosum*	NADH	184.7 ± 8.6
		NADPH	38.6 ± 2.2

200 µM of acetoacetyl-CoA and crude extracts of *S. cerevisiae* strains from overnight cultures cultivated in YNB-glucose were used. Averages and standard deviations were obtained from duplicate experiments

*ND* not detected

The expressed AAR from *A. vinosum* showed specific activity for both NADH and NADPH cofactors but it displayed 4.5-fold higher specific activity for NADH than for NADPH. In contrast the *C. necator* AAR showed significant activity for NADPH only. In addition, the NADPH-dependent activity was 4.3-fold higher for *A. vinosum* than for *C. necator* AAR.

Altogether the results demonstrated that the newly expressed AAR from *A. vinosum* was functionally expressed in *S. cerevisiae* and had a preference for NADH as cofactor.

### Aerobic PHB synthesis from xylose in shake flasks

**Fig. 3:**
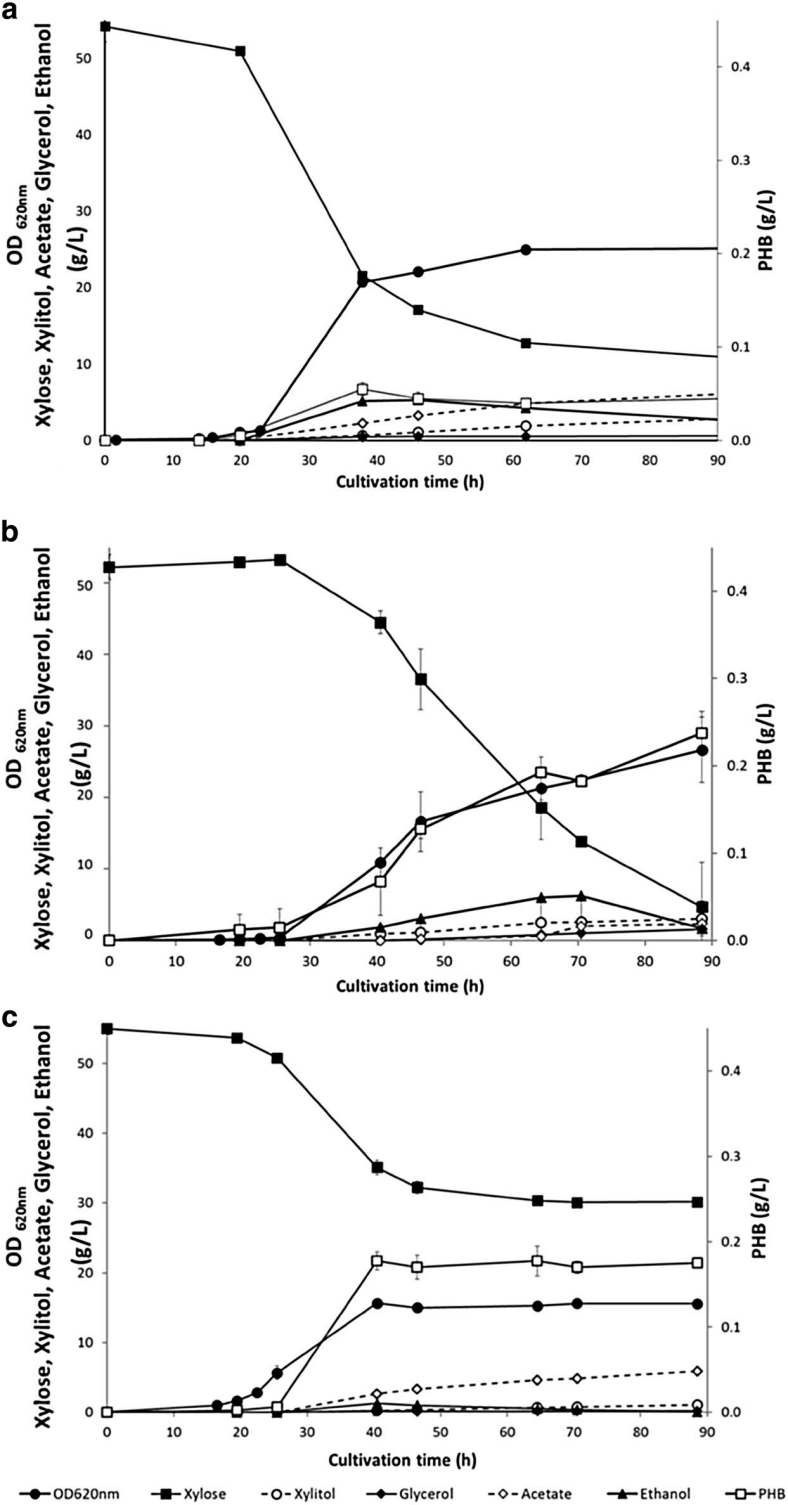
Aerobic growth and metabolite profiles from xylose for recombinant *S. cerevisiae* strains carrying a NAD(P)H-dependent PHB pathway. **a** TMB4443 (XR_wt_, CnAAR) [16] (rights and permission from AMB Express), **b** TMB4445 (XR_wt_, AvAAR) and **c** TMB4425 (XR_mut_, AvAAR). Strains were cultivated in biological duplicates on xylose in defined buffered medium using baffled shake flask. Results display a representative growth from two biological duplicates. *Error bars* represent standard deviation

The strains carrying the NADH-dependent AAR, i.e. TMB4425 (XR_mut_, AvAAR) and TMB4445 (XR_wt_, AvAAR), were studied under aerobic conditions in xylose using shake flasks, i.e. under the exact same conditions as the previously described strain TMB4443 (XR_wt_, CnAAR) [16]. Figure 3 shows a representative pattern for growth, xylose consumption, ethanol, acetate, glycerol formation and PHB production for the three strains. For comparison purposes, the control strains TMB4424 (XR_mut_) and TMB4444 (XR_wt_) that were previously cultivated under the same conditions [16], were also included in the result summary that is displayed in Additional file 2.

PHB production was detected during the exponential growth in all strains carrying a PHB pathway (Fig. 3). The results of the previously described strain that carries the XR_wt_ and the NADPH-dependent PHB pathway (TMB4443) are shown in Fig. 3a. In comparison, the newly generated strain harboring XR_wt_ and the NADH-dependent PHB pathway (TMB4445, Fig. 3b) displayed significantly higher PHB yield (4.3-fold), PHB titer (5.1-fold) and final PHB content per CDW (3.4-fold). When comparing strains carrying the NADH-dependent PHB pathway, PHB titers were 1.3-fold higher in TMB4445 (XR_wt_) than in TMB4425 (XR_mut_) but the final PHB content per CDW was 1.4-fold higher in TMB4425 than in TMB4445, as result of less xylose being used in the XR_mut_ strain (Fig. 3c; Additional file 2).

More generally strains harboring XR_wt_ (TMB4443, TMB4444, and TMB4445) had a higher growth rate than the ones carrying XR_mut_ (TMB4424, TMB4425). Xylitol was the main by-product from xylose fermentation and it was 2.5-fold higher in TMB4445 (XR_wt_) than in TMB4425 (XR_mut_). In both strains the glycerol yield remained low, while ethanol yields were lower in TMB4425 (XR_mut_) than in TMB4445 (XR_wt_) which correlates to a higher yield in acetate and PHB for TMB4425.

### Oxygen-limited cultivations

**Fig. 4:**
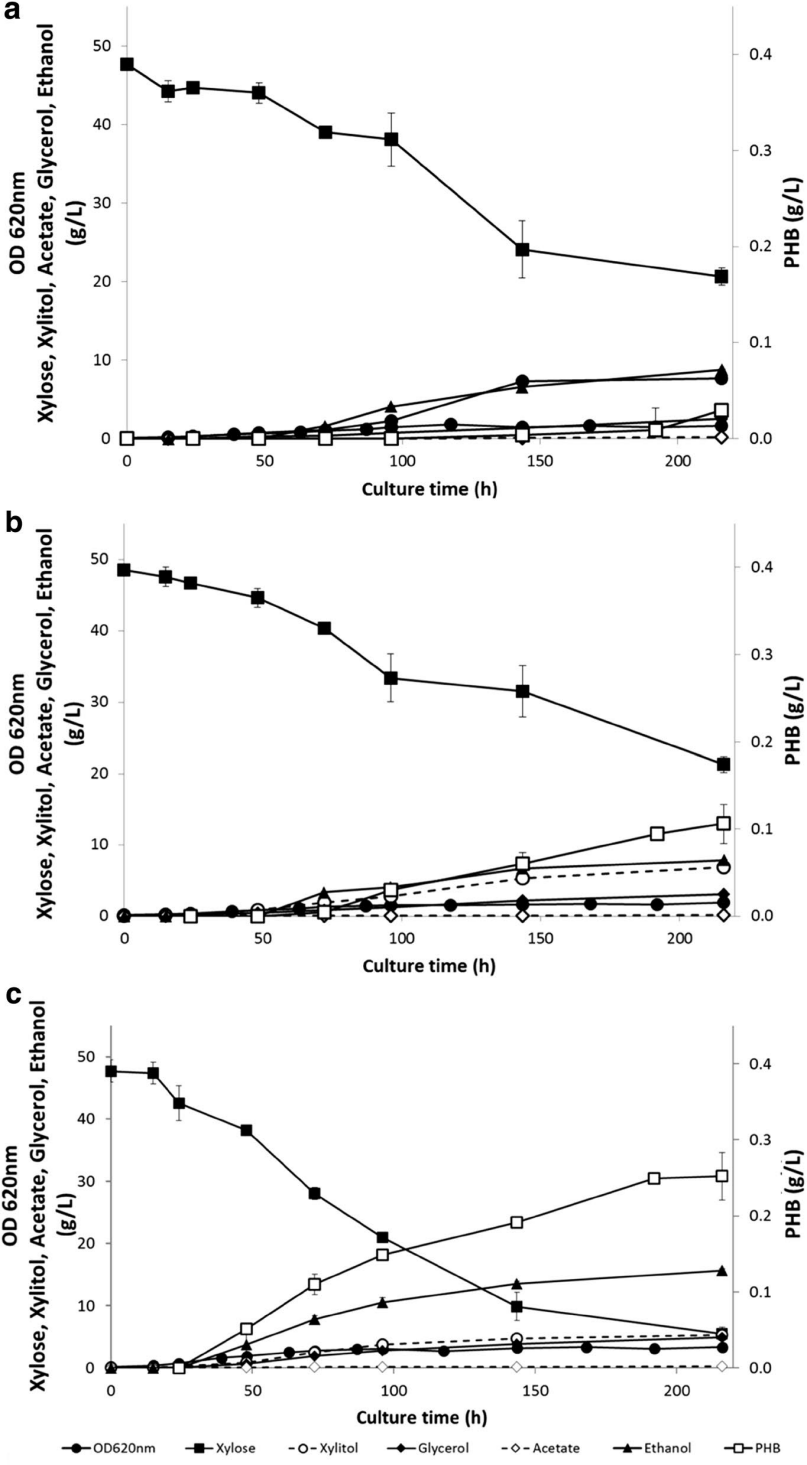
Oxygen-limited growth and metabolite profiles on xylose for recombinant *S. cerevisiae* strains carrying a PHB-pathway. **a** TMB4443 (XR_wt_, CnAAR). **b** TMB4445 (XR_wt_, AvAAR). **c** TMB4425 (XR_mut_, AvAAR). Strains were cultivated in biological duplicates on xylose in defined buffered medium using serum flasks. Results display a representative graph from two biological duplicates. *Error bars* represent standard deviation

To investigate the impact of the redox balance on PHB synthesis of the cell, cultivations were performed for the five strains under oxygen-limiting conditions using serum flasks (Additional file 2). Figure 4 shows the representative growth, xylose consumption, ethanol, acetate, glycerol formation and PHB during growth in oxygen-limited serum flasks for the three strains expressing a PHB pathway (TMB4443, TMB4445 and TMB4425).

TMB4425 (XR_mut_, AvAAR) reached the highest PHB titer, 252 mg/L, followed by TMB4445 (XR_wt_, AvAAR), 131 mg PHB/L, and TMB4443 (XR_wt_, CnAAR), 29 mg PHB/L. However, since the strains carrying XR_wt_ grew less efficiently on xylose under oxygen-limited conditions, the final PHB content per biomass was higher for TMB4445 (XR_wt_, AvAAR) (14.7%) than for TMB4425 (XR_mut_, AvAAR) (9.4%). In all cases, PHB was detected after 48 h of cultivation; also the strains showed higher biomass yields than their respective controls lacking the PHB pathway, which might be attributed to an increase of the cellular weight as a result of internal accumulation of PHB granules (Additional file 2).

At the metabolite level under oxygen-limiting conditions, the xylitol titer was higher for the strains expressing the XR_wt_, TMB4443 (7.63 ± 1.40 g) and TMB44445 (6.81 ± 0.09 g) than for the strain expressing the XR_mut_, TMB4425 (5.34 ± 0.15 g), which also correlates with their respective xylitol yields (0.28 ± 0.02; 0.25 ± 0.01 and 0.15 ± 0.01 g/g). This results confirmed what previous works showed about the importance of a more balanced co-factor ratio in the xylose metabolisation steps (see e.g. [31–33]). In particular, strains expressing XR_mut_ have higher affinity for NADH than XR_wt_ and therefore demand less usage of NADPH for xylose reduction thereby increasing the availability of NADPH for biomass formation and a cofactor balanced xylose conversion to xylulose [34].

Ethanol was produced and accumulated during exponential growth, but it was not re-assimilated. Final ethanol yields were slightly lower in the strain carrying XR_mut_ and the NADH-PHB pathway (TMB4425) than its respective control strain (TMB4424). Finally, glycerol and acetate levels remained low during all the cultivation time (Additional file 2).

### Anaerobic batch fermentations

**Fig. 5:**
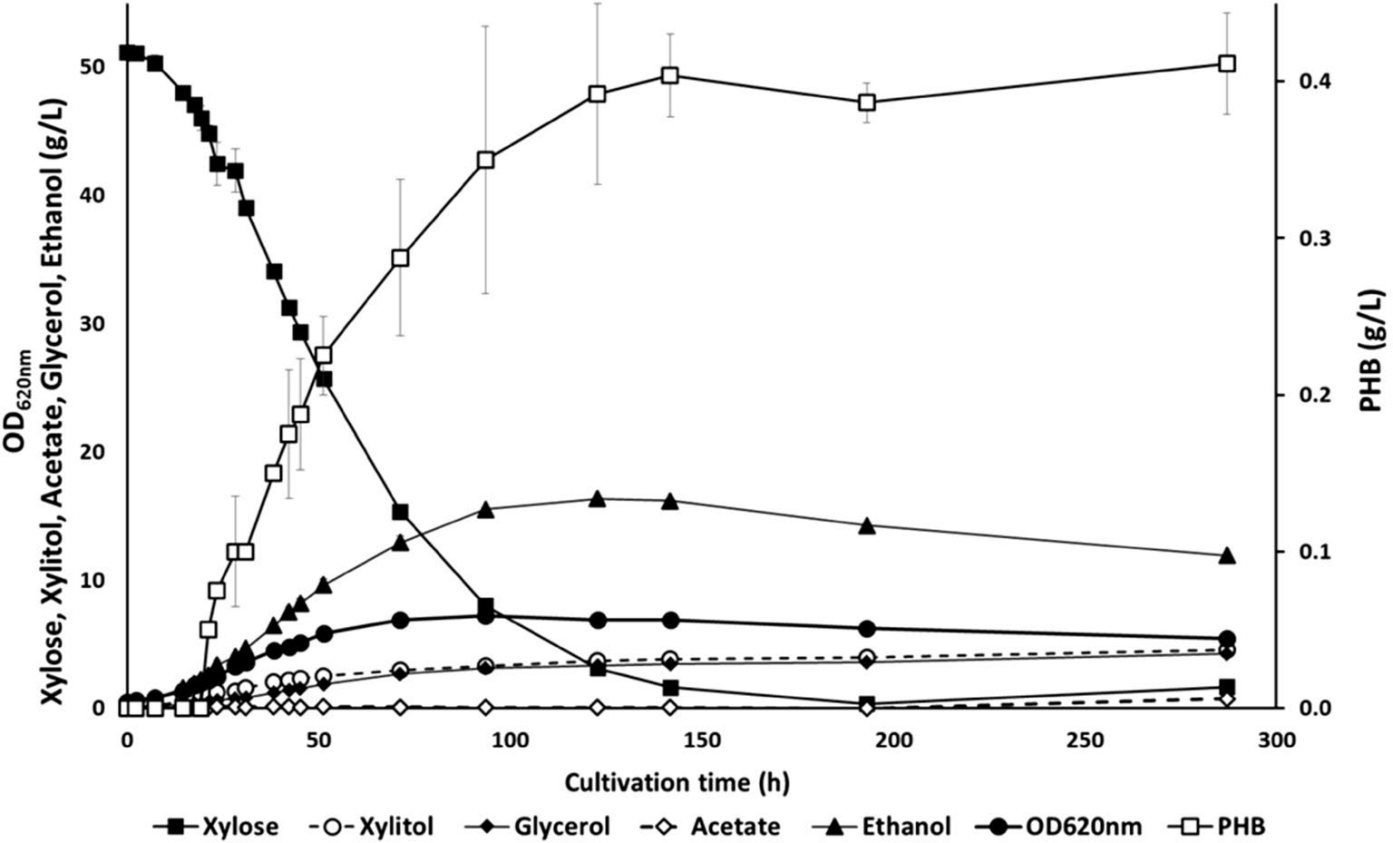
Representative anaerobic growth and metabolite profiles for recombinant *S. cerevisiae* TMB4425 (XR_mut_, AvAAR) strain on xylose defined medium at pH 5.5 using controlled bioreactors. *Error bars* represent standard deviation from two independent biological duplicates

The strain that displayed the most promising results in terms of PHB production, i.e. TMB4425 carrying a NADH-dependent PHB pathway and XR_mut_, was further evaluated in bioreactor under fully anaerobic conditions (Fig. 5).

TMB4425 grew at a maximum growth rate of 0.07/h, i.e. at similar values that those were previously obtained by Runquist and coworkers [20]. High xylose conversion efficiency was achieved, with only 2.1% of the total xylose remaining at the end of the fermentation. This resulted in a higher biomass level than under oxygen-limited conditions (Additional file 2). Ethanol was the most abundant metabolite, reaching a maximum titer of 16.4 g/L. After 150 h, 360 mg PHB/L was obtained, corresponding to a 1.4- and 2.0-fold increase in PHB production as compared to the oxygen-limited and aerobic cultivations, respectively. It also corresponded to the highest PHB content per biomass (14.2% of PHB of the total CDW) and the highest PHB yield per xylose consumed for this strain. The acetate, xylitol and glycerol yields were lower than under oxygen-limited conditions, suggesting that the carbon flux increased towards PHB production.

## Discussion

The present study proposes a metabolic engineering strategy to enhance the production of PHB from xylose in recombinant *S. cerevisiae*. The bacterial pathway for PHB production from *C. necator* has previously been integrated into xylose-utilising *S. cerevisiae* strains as part of a strategy to convert xylose into PHB [16]. Here we show that replacing the NADPH-dependent acetoacetyl-CoA reductase (AAR) from *C. necator* with the NADH-dependent AAR from *A. vinosum* is essential for boosting PHB production from xylose under aerobic and oxygen-limiting conditions. We also obtain anaerobic PHB production from xylose and the highest ever reported PHB content per cell in *S. cerevisiae*, in a strain combining the new AAR with a co-factor balanced xylose pathway. These results offer interesting insights for the potential of industrial anaerobic ethanol-PHB co-production from lignocellulosic feedstocks and other agricultural/industrial residues because during the anaerobic processes the metabolic flux for synthesis of ethanol and PHB is favored. In addition the need for sparging the reactor with air is no longer necessary, leading to a reduction of investments in equipment and energy supply that are necessary to increase oxygen transfer and cooling [35].

In native organisms PHB is a storage carbohydrate generated under starvation conditions, which implies that its production is growth-uncoupled [36]. This may explain why most PHB pathways can use NADPH-dependent AAR as it does not have to compete with the NADPH-demanding biosynthetic reactions. In *S. cerevisiae,* however, this is a drawback because the deregulated PHB pathway competes with biosynthesis for NADPH. Also, cytosolic availability of NADPH is known to be lower than for NADH [37]. Altogether this may contribute to the limited PHB yields that have been observed in this organism [13, 15, 16]. Using instead a NADH-dependent AAR should enable PHB production that is less dependent from biomass formation than with the regular *C. necator* pathway. Indeed, we were able to show that PHB volumetric titers, yields and PHB content per cell could be improved by using the NADH-dependent AAR from *A. vinosum*. It cannot be ruled out that the improvement may result from the overall higher NAD(P)H acetoacetyl-CoA conversion rate in the novel AAR. However the increasing PHB content per cell under oxygen-limiting conditions suggests that the availability of intracellular NADH plays a key role in the overall improvement.

A growth-coupled PHB production also implies that part of the cytosolic acetyl-CoA that is needed for biosynthesis has to be directed towards PHB formation. This can explain why PHB production is found to be inversely proportional to biomass formation and growth rate under all tested conditions. For example, TMB4425 carrying the NADH-favoring XR_mut_ has a lower growth rate than TMB4445 (carrying the NADPH-favoring XR_wt_) under aerobic conditions, as a result of limiting NADH availability under respiratory conditions, and it accumulates 1.4 times more PHB per cell; instead, when the oxygen availability is limiting, TMB4425 grows faster than TMB4445 (XR_wt_) due higher availability of NADH but the PHB content per cell is one-third lower than the one observed in TMB4445 (XR_wt_). More generally a reduction of the carbon flux towards biomass synthesis via the TCA cycle is expected to result in an increase in the flow towards ethanol and acetate, thereby increasing the levels of PHB precursors.

The choice of XR is also critical for anaerobic PHB production from xylose. We show that a combination of XR_mut_ and NADH-dependent AAR, i.e. TMB4425, should be used under these conditions to produce significant amounts of PHB because they favour NADH excess, which benefits both XR_mut_ and AvAAR activity, as well as a shift towards the fermentative pathway, thereby the PHB precursors, as a result of the absence of mitochondrial respiration. Still ethanol remains the major by-product of xylose fermentation, therefore it will be necessary to further increase the carbon flow towards the acetate and acetyl-CoA precursors, for instance by combining the current changes with the reported acetyl-CoA pool engineering strategies consisting in expressing heterologous pathways [38–41] or modifying the native pathways [39, 42–44].

Under oxygen-limiting conditions the strains carrying the PHB pathway (TMB4425 and TMB4445) were found to have higher glycerol yields and lower ethanol yields than the corresponding reference strains (TMB4424 and TMB4444). This can be expected when a part of acetaldehyde, i.e. the precursor of that contributes to the re-oxidation of NADH from glycolysis, is redirected towards acetate formation for PHB production. Indeed we observed that the difference in ethanol molar yield corresponded to the increase in glycerol molar yield (Additional file 3). This would mean that PHB synthesis cannot per se act as an alternative redox sink to ethanol, since its synthesis only regenerates 0.5 NAD^+^ per pyruvate, i.e. half of the NAD^+^ regenerated via ethanol formation. Therefore glycerol formation is preferred. We also hypothesize that the redirected flux from ethanol was used for PHB production; however the produced PHB level per g xylose was much lower than the potentially redirected acetyl-CoA precursor (Additional file 3); so a part of the redirected flux might be used for other purposes, which could for instance also explain the increase in biomass yield in the PHB-producing strains as compared to their controls.

## Conclusions

This work demonstrated the advantages of using a NADH-dependent AAR for PHB synthesis from xylose in *S. cerevisiae* compared to the regularly used NADPH-dependent AAR of *C. necator.* The NADH-dependent AAR from *A. vinosum* not only improved the conversion of xylose into PHB under aerobic conditions but it also enabled anaerobic PHB production from xylose when combined with redox engineered XR enzyme in recombinant *S. cerevisiae*. Further optimisation for higher titers, volumetric yields and productivities will notably require to engineer the central carbon metabolism in order to boost the production of the acetyl-CoA pathway precursor.

## Additional files

**Additional file 1.** Schematic representation of genes and regulatory sequences in the integrative vector YIpAGS3.

**Additional file 2.** The values reported are calculated from cultivations on defined media with xylose (50 g/L) as carbon source. The specific growth rate is calculated from the exponential phase. Yields and titers are calculated from a single time point in the end of the cultivation. Reported values represent the mean ± SD of at least two independent cultivations performed. Y_sX_: yield of biomass on xylose, Y_sEtOH_: yield of ethanol on xylose, Y_sAc_: yield of acetate on xylose, Y_sXylitol_: yield of xylitol on xylose, Y_sGly_: yield of glycerol on xylose, Y_sPHB_: yield of PHB on xylose. PHB titer: the volumetric PHB titer. PHB/CDW: The PHB component as percentage of total cell dry weight.

**Additional file 3.** Theoretical analysis of flux redirection in the case of TMB4425 under oxygen-limited conditions.

## Abbreviations

Acetyl-CoA: acetyl coenzyme A
AAR: acetyl-coA reductase
NAD^+^/H: nicotinamide adenine dinucleotide
NADP^+^/H: nicotinamide adenine dinucleotide phosphate
PCR: polymerase chain reaction
XDH: xylitol dehydrogenase
XR_mut_: mutated xylose reductase
XR_wt_: wild type xylose reductase
Y_sX_: yield of biomass on xylose
Y_sEtOH_: yield of ethanol on xylose
Y_sAc_: yield of acetate on xylose
Ys_Xylitol_: yield of xylitol on xylose
Y_sGly_: yield of glycerol on xylose
Y_sPHB_: yield of PHB on xylose
