# Supplementary material for: Anaerobic poly-3-d-hydroxybutyrate production from xylose in recombinant Saccharomyces cerevisiae using a NADH-dependent acetoacetyl-CoA reductase
Source: Microb Cell Fact. 2016 Nov 18;15:197. doi: 10.1186/s12934-016-0598-0 (PMC5116212; doi:10.1186/s12934-016-0598-0)
Supplement: Supplementary file 1 — Additional file 1. Schematic representation of genes and regulatory sequences in the integrative vector YIpAGS3. [file 12934_2016_598_MOESM1_ESM.docx]

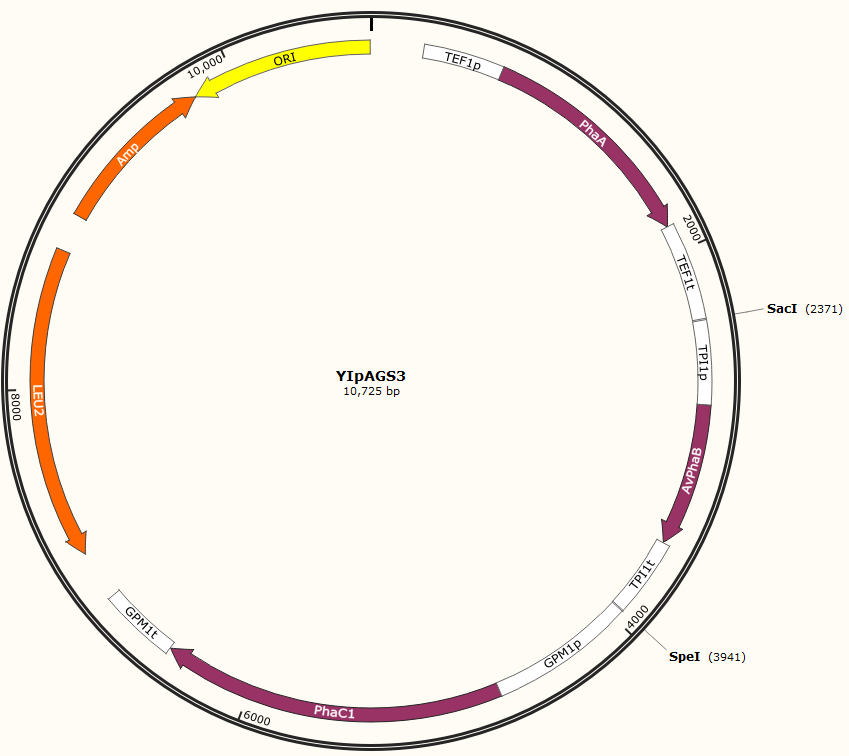
Schematic representation of genes and regulatory sequences in the integrative vector YIpAGS3.
