# Supplementary material for: Anaerobic poly-3-d-hydroxybutyrate production from xylose in recombinant Saccharomyces cerevisiae using a NADH-dependent acetoacetyl-CoA reductase
Source: Microb Cell Fact. 2016 Nov 18;15:197. doi: 10.1186/s12934-016-0598-0 (PMC5116212; doi:10.1186/s12934-016-0598-0)
Supplement: Supplementary file 2 — Additional file 2. The values reported are calculated from cultivations on defined media with xylose (50 g/L) as carbon source. The specific growth rate is calculated from the exponential phase. Yields and titers are calculated from a single time point in the end of the cultivation. Reported values represent the mean ± SD of at least two independent cultivations performed. YsX: yield of biomass on xylose, YsEtOH: yield of ethanol on xylose, YsAc: yield of acetate on xylose, YsXylitol: yield of xylitol on xylose, YsGly: yield of glycerol on xylose, YsPHB: yield of PHB on xylose. PHB titer: the volumetric PHB titer. PHB/CDW: The PHB component as percentage of total cell dry weight. [file 12934_2016_598_MOESM2_ESM.docx]

| **Strain** | **TMB4424** | **TMB4425** | **TMB4443** | **TMB4444** | **TMB4445** | **TMB4424** | **TMB4425** | **TMB 4443** | **TMB 4444** | **TMB4445** | **TMB4425** |
| --- | --- | --- | --- | --- | --- | --- | --- | --- | --- | --- | --- |
| **Relevant genotype** | XRmut | XRmut | XRwt | XRwt | XRwt | XRmut | XRmut | XRwt | XRwt | XRwt | XRmut |
|  |  | AvAAR | CnAAR |  | AvAAR |  | AvAAR | CnAAR |  | AvAAR | AvAAR |
| **Cultivation time (h)** | 82h | 82h | 82h | 82h | 82h | 216h | 216h | 216h | 216h | 216h | 144h |
| **Growth condition** | Aerobic | Aerobic | Aerobic | Aerobic | Aerobic | Oxygen-limited | Oxygen-limited | Oxygen-limited | Oxygen-limited | Oxygen-limited | Anaerobic |
| **Growth rate (h^-1^)** | 0.18 ± 0.05 | 0.16 ± 0.01 | 0.23 ± 0.01 | 0.25 ± 0.01 | 0.21 ± 0.02 | 0.07 ± 0.01 | 0.06 ± 0.00 | 0.04 ± 0.00 | 0.04 ± 0.00 | 0.04 ± 0.00 | 0.07 ± 0.00 |
| **Y_sX_ (g/g Xyl)** | 0.22 ± 0.02 | 0.15 ± 0.00 | 0.11 ± 0.02 | 0.14 ± 0.00 | 0.15 ± 0.02 | 0.04 ± 0.00 | 0.06 ± 0.00 | 0.03 ± 0.00 | 0.02 ± 0.01 | 0.03 ± 0.00 | 0.05 ± 0.00 |
| **Y_sEtOH_ (g/g Xyl)** | 0.04 ± 0.00 | 0.05 ± 0.00 | 0.10 ± 0.05 | 0.06 ± 0.00 | 0.11 ± 0.02 | 0.41 ± 0.00 | 0.37 ± 0.02 | 0.32 ± 0.01 | 0.31 ± 0.01 | 0.29 ± 0.02 | 0.32 ± 0.01 |
| **Y_sAc_ (g/g Xyl)** | 0.21 ± 0.04 | 0.24 ± 0.01 | 0.14 ± 0.00 | 0.14 ± 0.00 | 0.04 ± 0.03 | 0.00 ± 0.00 | 0.00 ± 0.00 | 0.01 ± 0.00 | 0.00 ± 0.00 | 0.01 ± 0.00 | 0.00 ± 0.00 |
| **Y_sXylitol_ (g/g Xyl)** | 0.04 ± 0.00 | 0.04 ± 0.00 | 0.05 ± 0.03 | 0.10 ± 0.00 | 0.10 ± 0.02 | 0.15 ± 0.01 | 0.13 ± 0.01 | 0.28 ± 0.02 | 0.31 ± 0.01 | 0.25 ± 0.01 | 0.07 ± 0.01 |
| **Y_sGly_ (g/g Xyl)** | 0.03 ± 0.03 | 0.01 ± 0.00 | 0.01 ± 0.00 | 0.02 ± 0.00 | 0.05 ± 0.02 | 0.03 ± 0.00 | 0.12 ± 0.01 | 0.09 ± 0.01 | 0.03 ± 0.00 | 0.11 ± 0.01 | 0.07 ± 0.00 |
| **Y_sPHB_ (mg PHB /g Xyl)** | 0.0 ± 0.0 | 7.2 ± 0.2 | 1.2 ± 0.2 | 0.0 ± 0.0 | 5.0 ± 0.3 | 0.00 ± 0.0 | 6.01 ± 1.15 | 1.08 ± 0.24 | 0.00 ± 0.00 | 4.80 ± 0.51 | 7.00 ± 1.60 |
| **PHB titer (mg/L)** | 0 ± 0 | 178 ± 4 | 45 ± 4 | 0 ± 0 | 238 ± 25 | 0 ± 0 | 252 ± 31 | 30 ± 9 | 0 ± 0 | 131 ± 23 | 360 ± 62 |
| **Final PHB content (%CDW)** | 0.0 ± 0.0 | 4.9 ± 0.0 | 1.0 ± 0.0 | 0.0 ± 0.0 | 3.4 ± 0.6 | 0.0 ± 0.0 | 9.5 ± 1.2 | 3.5 ± 1.1 | 0.0 ± 0.0 | 14.7 ± 2.6 | 14.2 ± 1.9 |
| **Xylose/sugar consumed (%)** | 49.8 ± 3.0 | 45.2 ± 1.5 | 76.8 ± 4.6 | 80.1 ± 3.0 | 90.8 ± 12.3 | 96.5 ± 1.1 | 88.4 ± 2.7 | 56.8 ± 6.7 | 84.5 ± 7.6 | 56.9 ± 1.9 | 97.9 ± 1.5 |
| **Reference** | This study | This study | [16] | [16] | This  study | This study | This study | This study | This study | This study | This study |

**Additional file 2. Physiological results of recombinant *S. cerevisiae* strains grown on xylose under different oxygen levels**

The values reported were calculated for cultivations on defined media with xylose (50 g/L) as carbon source. The specific growth rate is calculated from the exponential phase. Yields and titers are calculated from a single time point at the end of the cultivation. Reported values represent the mean ± SD of at least two independent cultivations performed. Y_sX_: yield of biomass on xylose, Y_sEtOH_: yield of ethanol on xylose, Y_sAc_: yield of acetate on xylose, Y_sXylitol_: yield of xylitol on xylose, Y_sGly_: yield of glycerol on xylose, Y_sPHB_: yield of PHB on xylose. PHB titer: the volumetric PHB titer. PHB/CDW: the PHB component as percentage of total cell dry weight.
