# Supplementary material for: Anaerobic poly-3-d-hydroxybutyrate production from xylose in recombinant Saccharomyces cerevisiae using a NADH-dependent acetoacetyl-CoA reductase
Source: Microb Cell Fact. 2016 Nov 18;15:197. doi: 10.1186/s12934-016-0598-0 (PMC5116212; doi:10.1186/s12934-016-0598-0)
Supplement: Supplementary file 3 — Additional file 3. Theoretical analysis of flux redirection in the case of TMB4425 under oxygen-limited conditions. [file 12934_2016_598_MOESM3_ESM.docx]

**Theoretical analysis of flux redirection in the case of TMB4425 under oxygen-limited conditions**

1. **NADH theoretically redirected from the ethanol step to the glycerol step**

Under oxygen-limited conditions, TMB4425 (carrying the PHB pathway) produced 0.09 g glycerol/ g of xylose less than the respective control TMB4424. This is equivalent to

$\frac{0.09 g{glycerol}/{g xylose}}{92.09\frac{g}{mol}}$ = 0.000977 mol glycerol/ g xylose (or 0.98 mmole glycerol/ g xylose)

As 1 mole of NADH is reoxidised per mole of glycerol or ethanol, it corresponds to a possible redirection of 0.98 mmole ethanol/ g xylose or 0.045 g ethanol/g xylose. This is indeed the measured ethanol yield difference (0.04 g ethanol/ g xylose).

1. **Additional PHB that can theoretically be generated from the redirected ethanol flux**

Up to 0.98 mmole ethanol/g xylose (theoretically-cf A) or 0.86 mmole ethanol/g xylose (experimentally) can be rechannelled towards acetate and PHB. This would correspond to 0.49 mmole PHB/g xylose or 0.43 mmole PHB/g xylose respectively (0.5 mole PHB/mole acetate).

In reality, the difference in PHB production is 0.006 g PHB/g xylose, which corresponds to

$\frac{0.006 mg{PHB}/{g xylose}}{192.034\frac{g}{mol}}$ = 0.03 mmole PHB/g xylose, i.e. much lower than the theoretically available flux above.
